# Personality Factors in Colorectal Cancer: A Systematic Review

**DOI:** 10.3389/fpsyg.2021.590320

**Published:** 2021-11-03

**Authors:** Federica Galli, Ludovica Scotto, Simona Ravenda, Maria Giulia Zampino, Gabriella Pravettoni, Ketti Mazzocco

**Affiliations:** ^1^Applied Research Division for Cognitive and Psychological Science, European Institute of Oncology, IRCCS, Milan, Italy; ^2^Department of Dynamic and Clinical Psychology, and Health Studies, Faculty of Medicine and Psychology, Sapienza University of Rome, Rome, Italy; ^3^Division of Gastrointestinal Medical Oncology and Neuroendocrine Tumors, European Institute of Oncology, IRCCS, Milan, Italy; ^4^Department of Oncology and Hemato-Oncology, University of Milan, Milan, Italy

**Keywords:** colorectal cancer, cancer risk, personality, character, behavior, cancer incidence, cancer onset

## Abstract

**Background:** The role of personality in cancer incidence and development has been studied for a long time. As colorectal cancer (CRC) is one of the most prevalent cancer types and linked with lifestyle habits, it is important to better understand its psychological correlates, in order to design a more specific prevention and intervention plan. The aim of this systematic review is to analyze all the studies investigating the role of personality in CRC incidence.

**Methods:** All studies on CRC and personality up to November 2020 were scrutinized according to the Cochrane Collaboration and the PRISMA statements. Selected studies were additionally evaluated for the Risk of Bias according to the Newcastle-Ottawa Scale (NOS).

**Results:** Eight studies met the inclusion criteria and were eventually included in this review. Two main constructs have been identified as potential contributors of CRC incidence: emotional regulation (anger) and relational style (egoism).

**Conclusion:** Strong conclusions regarding the influence of personality traits on the incidence of CRC are not possible, because of the small number and the heterogeneity of the selected studies. Further research is needed to understand the complexity of personality and its role in the incidence of CRC and the interaction with other valuable risk factors.

## Introduction

Colorectal Cancer (CRC), a tumor affecting the large intestine that includes the ascending, transverse, descending and rectal tract, is the third most commonly diagnosed malignancy and the second leading cause of cancer deaths, with approximately 1.9 million new cases and 935,000 deaths worldwide reported in 2020, according to World Health Organization Globocan database (Sung et al., 2021). The 1-year survival rate is about 80% after diagnosis. The 5-year rate is between 45 and 65% in developed countries and between 8 and 45% in developing countries (Keum and Giovannucci, 2019). Industrialization and economic growth have worsened the situation, promoting a sedentary lifestyle, poor dietary habits, alcohol consumption and smoking (American Psychiatric Association, 2013; Murphy et al., 2019). Such behavioral factors, together with environmental and genetic factors are considered among the major risk factors for CRC development (Murphy et al., 2019). The impact of psychological factors on the incidence of CRC has been studied including anxiety and depression (Kroenke et al., 2005) and perceived stress (Kikuchi et al., 2017). Such studies are coherent with paradigms that consider psychological components and personality characteristics as factors affecting mortality (Roberts et al., 2007; O'Súilleabháin et al., 2021). According to recent works, psychological traits seem to share a common pathogenic mechanism typical of increased mucosal inflammation, metabolic parameters and proinflammatory status (Mancini et al., 2020). In line with this, an increasing number of studies are equating psychosocial factors (depression, anxiety, hostility, social isolation) to biological factors (smoking, hypercholesterolemia, hypertension, obesity, diabetes) in the pathogenesis of several diseases (Attilio et al., 2018). In the oncological field, a meta-analysis carried out on 165 controlled studies showed that stress-prone personality or unfavorable coping styles and negative emotional responses are related to an increased incidence of cancer, a worse prognosis and an increase in mortality (Chida et al., 2008).

Considering the multifactorial characterization of CRC, the aim of the present work was to investigate the psychological factors that may affect the incidence of CRC. Some studies (Schoormans et al., 2017; Lloyd et al., 2019; Coker et al., 2020) investigated personality characteristics either as outcomes of diagnosis and oncological treatments or as predictors of recovery from cancer. Fewer studies investigated the contribution of such characteristics on the incidence of CRC.

Personality can be defined as “relatively stable ways of thinking, feeling, behaving, and relating to others” (Lingiardi and McWilliams, 2017). Coherently with such a definition, expression of personality can be found in specific actions that allows the individual to protect him or herself from emotional hazards and to adaptively connect with the environment and with others. These protective and adaptive actions move along a continuum from full awareness to unconsciousness, with deliberative behaviors on one extreme, defensive mechanisms (Butow et al., 2000; Drageset and Lindstrøm, 2003; Beresford et al., 2006; Chida et al., 2008; Perry et al., 2015), on the other extreme and coping style (Neeleman et al., 2004; Perry et al., 2015) in between. In this perspective, personality denotes the kind of adaptation that individuals make to the external environment, including life-styles (Sutin et al., 2019), and health related behaviors. Similarly, personality features such as conscientiousness and neuroticism (Nakaya et al., 2010; Grov and Dahl, 2020) have been related to obesity (Mills et al., 2018) and dietary habits (Lutgendorf and Sood, 2011). If personality is related to behaviors, behaviors seem to be related to diseases. In particular, behavioral and life-style patterns as dietary habits, physical inactivity, obesity and abdominal fat, smoking and alcohol consumption have been associated with the incidence of diseases such as CRC (Murphy et al., 2019). Such personality and behavioral factors may influence cancer development and progression through mechanisms such as cellular immune response, oxidative stress, invasion, angiogenesis and inflammation (Jaffe, 2013; Di Giuseppe et al., 2018; O'Súilleabháin et al., 2021). However, studies investigating premorbid common traits in individuals developing any type of cancer showed controversial results: some large-scale cohort studies found no association between personality and cancer incidence (Temoshok, 1987; Jokela et al., 2014), while other studies did (Lemogne et al., 2013; Dong and Jin, 2018). In another study, Type C (or cancer prone personality, that implies the suppression of feeling and/or expression of negative emotions, focus on others' needs more than on one's own, unassertiveness, cooperation and acceptance) (Wellisch and Yager, 1983) and Type D (or depressed that means negative affectivity and social inhibition) personalities have been the object of investigation. More specifically, according to Temoshok (1987), Type C personality is associated with the weakening of the immune system and consequently the risk of developing cancer. Other authors hypothesized that reactions of helplessness and hopelessness to stressors are predictive of a worse outcome in breast cancer patients (Greer et al., 1979) or incidence of a new cancer in individuals (Greer and Watson, 1985). However, as Wellisch and Yager (1983) argued, not all types of cancer are the same and searching for common personality traits in individuals with different types of cancer could obscure features related to some specific sub-types. In the case of CRC, several studies investigated the role of personality on specific outcomes following a cancer diagnosis and treatment. For example, there is evidence of the role of personality characteristics [such as emotional lability, extraversion, openness to experience, agreeableness, conscientiousness (Paika et al., 2010), denial and sense of coherence (Hyphantis et al., 2011), repression and sense of coherence (Glavić et al., 2014) or neuroticism (Ristvedt and Trinkaus, 2005)] in predicting Quality of Life (QoL) and illness perception (Mols et al., 2012a; Schoormans et al., 2017) after cancer and related treatments. In other studies, Type D Personality and its components (negative affectivity and social inhibition) were associated with all-cause mortality (Shun et al., 2011), worse QoL in different domains (Shun et al., 2011; Mols et al., 2012b; Husson et al., 2015; Zhang et al., 2016) and most disease-specific symptoms (Zhang et al., 2016) in CRC patients. Beside the importance of personality in coping with cancer after a diagnosis, this review wants to focus on personality as a contributing risk factor for the incidence of CRC. Indeed, recent studies illustrated the need to consider also psychological and behavioral factors, besides genetic and environmental factors, to obtain a more comprehensive picture of the determinants underlying the incidence of CRC. Moreover, as personality refers to stable characteristics that affect individuals' behavior across different life domains, we were interested to investigate whether specific personality patterns are more likely to be associated with CRC incidence. To the best of our knowledge, a systematic review on personality features related to the incidence of CRC has never been realized.

## Methods

### Search Strategy

The procedure has been conducted according to the PRISMA statement (Moher et al., 2009). To include the broadest range of relevant literature, an electronic search was conducted on the major databases in the field of health and social sciences: Pubmed, Scopus, Embase, PsycInfo, Ovid, and Web of Science. The search was performed using Mesh terms/Keywords (depending on the database) with the same search strategy: “Colorectal neoplasm,” “Colon-Rectal Cancer” OR “Colorectal cancer” OR “Rectal cancer” OR “Colon cancer” OR “bowel cancer” AND “Personality” OR “Personality disorder” OR “Temperament” OR “Character.” The use of Mesh terms further guaranteed the complete description of any aspect of the main subject (personality). Our definition of personality pertains to a continuum, ranging from healthy (personality traits and styles, character, temperament) to the pathologic (e.g., personality disorders) in order to catch the widest number of papers on the topic. The choice of the keywords reflects the wide range definition of personality that we adopted.

The search was limited to papers in English published up to November 30th 2020. An additional analysis of the reference list of each selected paper was also performed. If the full text was not retrievable, the study was excluded.

### Inclusion Criteria

The following inclusion criteria were adopted in the articles' selection phase: (1) Studies with an analytical study design as defined by Grimes and Schulz (2002), in particular prospective and retrospective, case-control, longitudinal, cohort studies, randomized and clinical trials; (2) studies concerning the incidence of CRC with a confirmed histology, without time limits after diagnosis; (3) studies published in English.

### Exclusion Criteria

The following exclusion criteria were adopted: (1) studies that evaluated the role of personality on patients' outcomes after diagnosis and treatment; (2) articles investigating the contribution of personality on recurrence and on cancer survivorship; (3) studies based on animal models; (4) letters, commentaries, editorials, case reports, conference papers, book chapters, reviews, meta-analyses; (5) Self-reported diagnosis of CRC; (6) Correlational studies (also cohort studies without comparison/control group(s) have not been included); (7) Number of subjects per group ≤ 20 to have a stronger statistical power.

### Data Extraction

Study selection was performed by two independent reviewers with research expertise in general and clinical psychology (FG and LS) who assessed the relevance of the study for the objectives of this review. After the initial step of identification of records through different databases and duplicate removal, the following phase (screening) was the selection of papers on the base of title, abstract, and keywords of each study. The full text was retrieved, if the reviewers did not reach a consensus or the abstract did not contain sufficient information.

In the phase of eligibility, all full-texts were retrieved and a final check was made to exclude papers not responding to inclusion/exclusion criteria. A final consensus to decide the inclusion in the final selection was taken.

**Figure 1:**
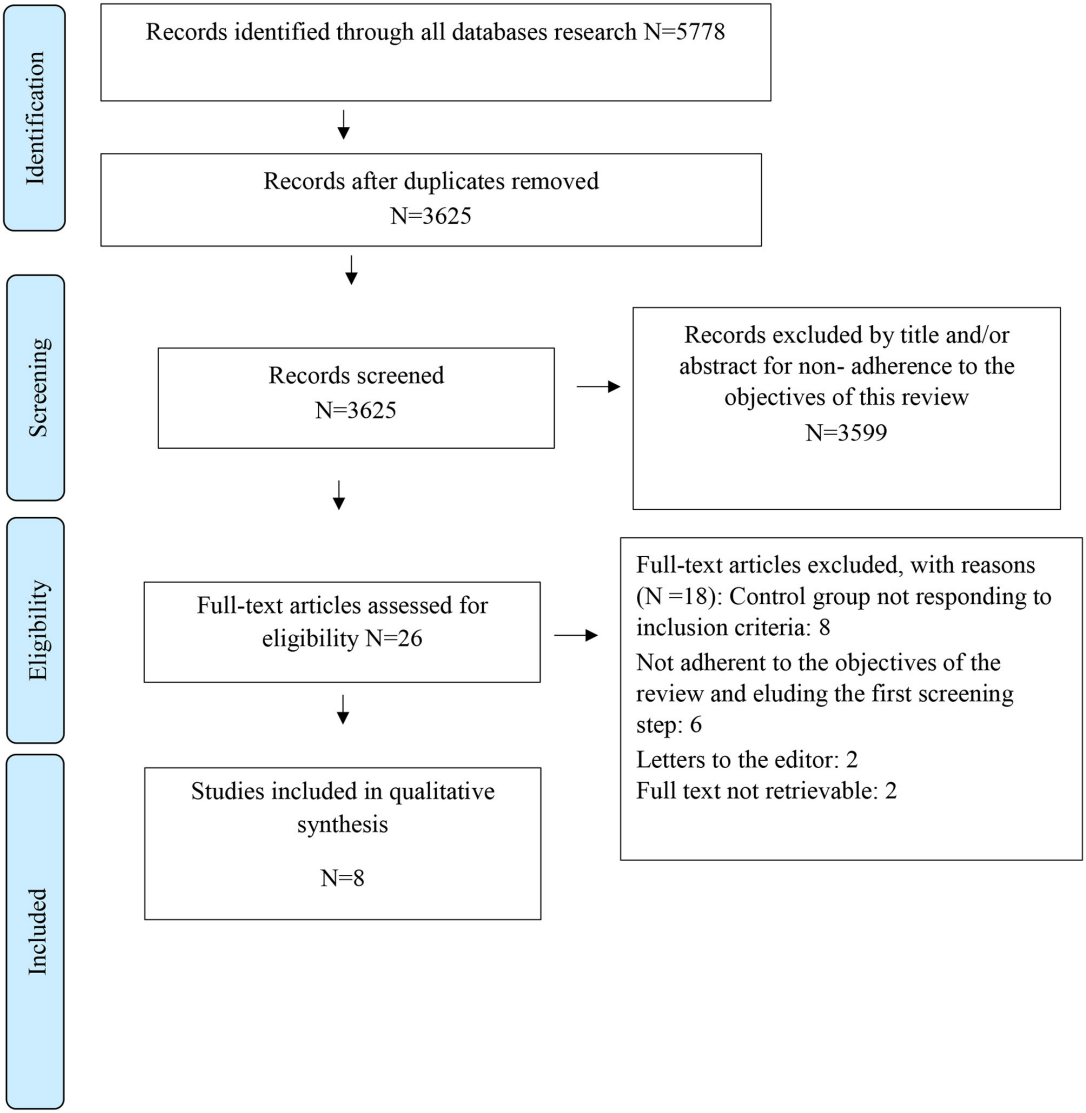
PRISMA flow diagram of literature search and selection of publications.

A standardized data extraction form was prepared; data were independently extracted by two of the authors (FG and LS) and inserted into a study database. Discrepancies between reviewers were resolved by a process of discussion/consensus moderated by a third reviewer (KM) (Furlan et al., 2009) (Figure 1).

### Statistical Methods

A systematic analysis was conducted according to the Cochrane Collaboration guidelines (Higgins and Green, 2011) and the PRISMA Statement (Moher et al., 2009). It was not considered appropriate to undertake a meta-analysis as the included studies were highly heterogeneous in terms of variables, instruments and outcomes (Higgins and Green, 2011).

### Risk of Bias

Quality assessment of each of the included studies was evaluated following the Newcastle-Ottawa Scale (NOS) for case-control studies on a 9-star model (Wells et al., 2014) (**Table 2**). The Newcastle-Ottawa Scale quality instrument is scored by awarding a point for each question in relation to the following categories: selection comparability and outcome. Possible total scoring is four points for Selection, two points for Comparability, and three points for Outcomes. Two reviewers (LS and FG) independently extracted relevant information and data from all eligible studies according to the appropriate inclusion criteria. Studies scoring above the median NOS value were considered as high quality (low risk of bias) and those scoring below the median value were considered as low quality (high risk of bias).

#### Characteristics of Study Populations

The overall number of CRC patients in the examined studies was 3,105 (Table 1). CRC patients were at different disease stages and/or treatment stages (undergoing/undergone surgery, chemotherapy, radiotherapy). Healthy controls were 75,097 while other cancer controls (prostate cancer, breast, lung or smoking-related cancer) were 8,128 (Table 1).

**Table 1:** Personality and colorectal cancer: description of the selected studies.

**References**	**Study design**	**Population**	**Psychological assessment tools**	**Personality outcome**	**Timing of measurement**	**Main results**	**Note**
Lemogne et al. (2013)	Cohort study	124 CRC (106m, 18f); 368 prostate cancer, 146 breast cancer, 137 smoking related cancers (132M, 5F), 352 other cancers (256m, 96f) Country: France	- Personality Stress Inventory - Buss and Durkee Hostility Inventory - Bortner Type A Rating Scale	Type 1(suppress negative emotions), Type 5 personalities (rational anti-emotional), Hostility, Type A behavior pattern (time urgency, competitiveness, need for achievement)	Personality questionnaires: at baseline Cancer incidence: retrieved through annual follow-ups (maximum 16-year follow-up from baseline)	No association between Type 1 (suppress negative emotions) and CRC No association between Type 5 (rational anti-emotional) and CRC No association between Hostility and CRC No association between Type A behavior pattern (time urgency, competitiveness, need for achievement)	- Risk of false negative cases. - Mailed questionnaire
Nakaya et al. (2010)	Cohort study	376 CRC, 180 stomach, 364 lung, 908 breast, 105 liver, 140 pancreas, 113 cervix uteri, 138 corpus uteri, 479 prostate, 122 kidney, 172 urinary organs, 170 melanoma, 220 nervous system Country: Finland and Sweden	Eysenck Personality Inventory	Extraversion, Neuroticism	Personality Questionnaire: at baseline Cancer incidence: national tumor registry with a maximum follow-up of 30 years	No association between personality traits and CRC incidence	- Delivered questionnaire
Nagano et al. (2008)	Case-control study	497 CRC (288 m, 209 f), 809 healthy controls (502 m, 307 f) Mean age for CRC: 59 yrs Country: Fukuoka (Japan)	Stress Inventory	Emotional suppression (unfulfilled needs for acceptance); Loss-hopelessness (Low sense of control, Object-dependence/loss, Object-dependence/happiness); Hysterical personality (“object dependence/ambivalence” and “egoism”).	Personality questionnaires: at the histologically confirmation of diagnosis (before or after surgery) Healthy controls: before or after surgery	Weak positive association between object-dependence/happiness and CRC (*p* = 0.05). Negative association between hysterical personality (object-dependence/ambivalence (*p* = 0.04) and egoism (*p* = 0.01) and CRC risk.	- The reason for the selection of some sub-scales is unclear. - The selection of items for Hysterical personality is weak. - Participation rate higher for cases (74%) than controls (59%).
Kreitler et al. (2008)	Case-control study	230 CRC (: 101 m 129 f); 165 healthy (55 m 110 f ), 90 Crohn's disease (49 m, 41 f) M. Age for CRC: 61.54	Cognitive Orientation Questionnaire	Beliefs	Patients: at the time of treatment or follow-up Healthy controls: in their working place	Positive association between Cognitive Orientation and CRC (*p* < 0.001): - Positive association between Conflict in self-effacement vs. self-assertion - Positive association between Conflict in closeness to others vs. distancing from others	- the methodology is poorly described - the factors measured by the CO questionnaire include a variety of different themes - difficult to say if the beliefs are the cause or the effect of cancer.
White et al. (2007)	Cohort Study	280 CRC, 352 breast, 318 prostate, 88 lung, 261 melanoma, 653 other Range: 27–75 years, M. Age - CRC: 61.8 Country: Melbourne (Australia)	7-item anger control subscale (from Courtauld Emotional Control Scale); Positive and Negative Affect Scale	Anger control, Negative affect	Baseline (healthy population) Average follow-up of 9 years	- -Positive association between anger control and presence of CRC; - Positive association between Negative affect and presence of CRC - weak positive associations between negative affect and colorectal cancer after adjusting for risk factors (the association between anger control and colorectal cancer was slightly stronger, excluding the first 2 years of follow-up).	- the measurement of negative affect and anger control might not be stable over several years.
Nakaya et al. (2003)	Cohort study	186 CRC, 229 stomach, 108 lung, 87 breast Country: rural northern Japan	Eysenck Personality Questionnaire -Revised Short Form	Extraversion, Neuroticism, Psychoticism	7 year follow-up (for prospective design)	No association with CRC	- Delivered questionnaire - the number of cases of cancer for single site is modest and the statistical power might not be sufficient. - Neuroticism showed significant association at 3 year but not 7 year follow-up for all cancer site incidence.
Kavan et al. (1995)	Case-matched control study	61 CRC veterans; 61 healthy veterans, 100% males M. age: na Country: Minneapolis (USA)	21-factor Minnesota Multiphasic Personality Inventory	Aggressive hostility, Psychoticism-Peculiar Thinking, Cynicism-Normal Paranoia, Stereotypic Femininity and Masculinity, Psychotic Paranoia, Assertiveness, Intellectual Interests, Dreaming, Denial of Somatic Problems, Neurasthenic somatization, Sexual adjustment, Well-being – Health, Family Attachment, Social extraversion, Delinquency, Inner Directedness, Religious Fundamentalism, Phobias, Neuroticism Depression	5–37 years (M = 20.5) premorbid assessment Healthy controls: information non-specified	- Positive association between aggressive hostility (*p* < 0.018) and CRC incidence; - Positive association between phobias (*p* < 0.05) and CRC stage of presentation - Negative association between religious fundamentalism (*p* < 0.05) and CRC stage of presentation - No association in the other subscales	Premorbid assessment further supports findings
Kune et al. (1991)	Case-matched control study	637 CRC (346 m, 291 f); 714 healthy controls (391 m, 323 f); Mean age: 65 Yrs. Country: Melbourne (Australia)	Questionnaire on cancer-prone personality	Commitment to conformity with social norms, Negative emotions repression or denial, suppression	Not reported	- combined score for cancer personality questions (commitment to conformity with social norms, negative emotions repression or denial, suppression, low anxiety, conflict avoidance) differed significantly (*p* < 0.001) - positive association between anger subscale (repression, denial, non-expression of anger) and CRC (*p* < 0.001) - stronger positive association between denial of anger and CRC in women (*p* = 0.005) - Positive association between Commitment to conformity with social norms and CRC in women (*p* = 0.005)	- Risk of recall bias - Questionnaire structured ad hoc (no validation against other measures) - 5% of cases did not know if they were affected by CRC

*CRC, colorectal cancer; ICD-9, International Classification of Diseases*.

## Results

Eight studies were included in the final qualitative analysis. Table 1 reports the details of all the selected studies (Kune et al., 1991; Kavan et al., 1995; Nakaya et al., 2003, 2010; White et al., 2007; Kreitler et al., 2008; Nagano et al., 2008; Lemogne et al., 2013).

### Characteristics of Reviewed Studies

#### Psychological Assessment Tools

All the selected studies adopted differing instruments of assessment (see Table 1): questionnaires on cancer-prone personality (Questionnaire on cancer-prone personality) (Kune et al., 1991), questionnaires grounded on specific theories as the Personality Stress Inventory (Lemogne et al., 2013), the Stress Inventory (Nagano et al., 2008), the Buss and Durkee Hostility Inventory (Lemogne et al., 2013), the Bortner Type A Rating Scale (Lemogne et al., 2013), the Cognitive Orientation Questionnaire (Kreitler et al., 2008), the Courtauld Emotional Control Scale (White et al., 2007) or more acknowledged ones as the Positive and Negative Affect Scale (White et al., 2007) for the evaluation of negative or positive affect, the Minnesota Multiphasic Personality Inventory (Kavan et al., 1995) and the Eysenck Personality Inventory (Nakaya et al., 2003, 2010) for the assessment of personality.

#### Timing of Assessment

The follow-up period ranged from 0 (coincident with the moment of CRC diagnosis) to 37 years.

#### Research Design

Out of the eight studies, four adopted a case-control design (Kune et al., 1991; Kavan et al., 1995; Kreitler et al., 2008; Nagano et al., 2008) while the remaining four were cohort studies (Nakaya et al., 2003, 2010; White et al., 2007; Lemogne et al., 2013).

#### Case-Control Studies

The study by Kavan et al. (1995) was conducted on a sample of 124 male veterans living in Minneapolis (USA) who completed the personality questionnaire (MMPI) between 1947 and 1975. Among those, 61 were identified through the regional tumor registry as having had a colon cancer diagnosis in the years between 1977 and 1988. The interval between the MMPI completion and colon cancer diagnosis ranged from 5 to 37 years. The 61 control cases were matched for age, education and source of referral for medical care.

Nagano et al. (Nagano et al., 2008) recruited 497 newly diagnosed CRC patients (age: 20–74) from 8 large hospitals in the Fukuoka area of Japan and 809 controls (age: 20–74) matched by gender and age and randomly selected in the same community area. A survey was performed by a research nurse during which questionnaires on personality and health habits were administered before or after surgery for cases and controls.

In Kreitler et al.'s study (Kreitler et al., 2008) 230 patients with CRC (mean age: 61.54) were compared with 90 patients with a confirmed diagnosis of Crohn's disease and with 165 healthy controls. The proportions of men in the three groups are were 33.3, 50.6, and 43.9%, respectively. No other information on the sample and on recruiting methods was reported.

The case-control study by Kune et al. (1991) involved 637 patients (346 males and 291 females) with first diagnosis of CRC between April 1980 and April 1981 and resident in Melbourne. Seven hundred and fourteen controls (391 males and 323 females) were randomly selected from the Community of the Metropolitan Melbourne area, and matched with cases according to age and gender.

#### Cohort Studies

Nakaya et al. (2010) conducted a prospective study based on combined data of Swedish twins and singletons (*N* = 29,828; age range: <20–49) born before 1958 and that responded to the baseline questionnaire in 1973 and of Finnish twins and singletons (*N* = 29,720; age range: <20–>70) born before 1958 and that responded to the baseline questionnaire in 1975. Information on cancer diagnoses was obtained by record linkage to the national cancer registries in Finland and Sweden, both holding information on all cases of cancer diagnosed since 1958. Personality was measured at baseline, together with socio-demographic information and health habits. Information on cancer diagnosis was retrieved by record linkage to the national cancer registries in Finland and Sweden with a maximum 30-year follow-up. Another study conducted by Nakaya et al. (2003) investigated the role of personality on cancer risk in 30,277 persons (age range: 40–64) living in northern Japan from June through August 1990. Questionnaires on personality and health habits were collected at the participants' residences by the health promotion committees appointed by the government. Information on cancer incidence was collected through the linkage with the Prefectural Cancer Registry that covers the study area, for a maximum of 7-years follow-up. Lemogne et al. (2013) conducted a cohort study on a target population consisting of 44,992 employees of the French national gas and electricity company. Among those, 20,625 employees (45.8%) (15,011 men and 5,614 women) volunteered to participate and 20,488 completed the survey. Among these, personality measures were available at baseline for 13,768 participants. Annual follow-ups to check for diagnosis of primary cancer were conducted from 1994 to 2009. Diagnoses self-reported by patients were verified through the French national cause-of-death registry.

The study by White et al. (2007) included a cohort of 19,730 participants (aged range: 27–75) recruited from 1990 to 1994 in Melbourne, Australia. Participants completed a questionnaire on personality, health habits, biological and demographic variables in an assessment center. Physical assessments (e.g., blood test, height, weight) were also performed. Cancer diagnoses were identified by record linkage to the State Cancer Registry during the follow-up period (average follow-up: 9 years).

#### Risk of Bias

Five studies were quoted as high quality (low risk of bias) and three were low quality (high risk of bias) by NOS (see Table 2).

**Table 2:** Risk of bias.

**References**	**Selection**				**Comparability^a^**	**Outcome (psychological tests)**			**Total NOS score**
	**Adequate case definition**	**Representativeness**	**Selection of controls**	**Definition of controls**		**Ascertainment**	**Same Ascertainment for case/control**	**Non-response rate**	
Lemogne et al. (2013)	–	*	*	*	–	–	*	–	4/9
Nakaya et al. (2010)	*	*	*	–	**	*	*	*	8/9
Nagano et al. (2008)	*	*	*	*	**	*	*	*	9/9
Kreitler et al. (2008)	–	–	–	–	*	*	*	–	3/9
White et al. (2007)	*	*	*	–	*	–	*	*	6/9
Nakaya et al. (2003)	–	–	*	–	**	–	*	–	4/9
Kavan et al. (1995)	*	–	*	*	**	*	*	*	8/9
Kune et al. (1991)	*	*	*	–	**	–	*	–	6/9

a *One star was assigned when the criterion was fulfilled by that study. Two stars were assigned when the control was matched both for age and other controlled factor(s)*.

*The 8 criteria were operationalized using the Newcastle-Ottawa Scale (NOS) (Wells et al., 2014)*.

### Narrative Synthesis on Personality Characteristics

Relative to the specific personality characteristics, the studies presented a high heterogeneity in the type of characteristics assessed and in the assessment measures (see Table 1). We divided them into 4 main categories: emotional regulation, cognitive schemata, body-related processes and relational style (see Table 3).

**Table 3:** Personality factors association or not with CRC incidence in the selected studies.

	**Association with CRC incidence**	**No association with CRC incidence**
Emotional regulation	Phobias (Kavan et al., 1995) - PA Aggressive Hostility (Kavan et al., 1995) - PA Anger control/ negative affect (White et al., 2007) – PA (after adjusting for risk factors) Anger repression, Anger denial, non-expression of anger (Kune et al., 1991) - PA Denial of anger in women (Kune et al., 1991) - PA	Hostility (Lemogne et al., 2013) Type 1 (suppress negative emotions) (Lemogne et al., 2013) Extraversion (Nakaya et al., 2003, 2010) Neuroticism (Kavan et al., 1995; Nakaya et al., 2003, 2010) Emotional suppression: Unfulfilled needs for acceptance, Altruism, Rationalizing conflicts/frustrations (Nagano et al., 2008) Depression (Kavan et al., 1995)
Cognitive schemata	Religious Fundamentalism (Kavan et al., 1995) – NA	Type 5 (rational anti-emotional) (Lemogne et al., 2013) Psychoticism (Nakaya et al., 2003) Psychoticism-Peculiar Thinking (Kavan et al., 1995) Cynicism-Normal Paranoia (Kavan et al., 1995) Stereotypic Femininity and Masculinity (Kavan et al., 1995) Psychotic Paranoia (Kavan et al., 1995) Assertiveness (Kavan et al., 1995) Intellectual Interests (Kavan et al., 1995) Dreaming (Kavan et al., 1995)
Body-related processes		Denial of Somatic Problems (Kavan et al., 1995) Neurasthenic somatization (Kavan et al., 1995) Sexual adjustment (Kavan et al., 1995) Well-being – Health (Kavan et al., 1995)
Relational style	Loss-hopelessness: Object-dependence/happiness (Nagano et al., 2008) – PA Hysterical personality: Egoism and object-dependence/ambivalence (Nagano et al., 2008) – NA Beliefs: conflict in self-effacement vs. self-assertion (Kreitler et al., 2008) – PA Beliefs: conflict in closeness to others vs. distancing from others (Kreitler et al., 2008) - PA Perfect duty performance (Kreitler et al., 2008) - PA Commitment to conformity to social norms (Kune et al., 1991) - PA	Extroversion (Nakaya et al., 2003) Type A behavior pattern (time urgency, competitiveness, need for achievement) (Lemogne et al., 2013) Family Attachment (Kavan et al., 1995) Social extraversion (Kavan et al., 1995) Delinquency (Kavan et al., 1995) Inner Directedness (Kavan et al., 1995)

*PA, Positive Association; NA, Negative Association*.

#### Emotional Regulation

ER refers to the processes that individuals use to identify which emotions they have, when they have them, and how these emotions are experienced and expressed (Gross, 2002). Two studies (Kavan et al., 1995; Lemogne et al., 2013) investigating the role of Hostility, showed opposing results. More specifically, Kavan et al. (1995) found that the two factors that significantly discriminated between CRC patients and healthy controls, among those measured by the MMPI, were Phobias and Aggressive Hostility, with a higher level of these variables in CRC patients compared to healthy subjects. On the contrary, Lemogne et al. (2013) did not find any association between incidence of CRC and Hostility and Type 1 (suppress negative emotions). However, it is worth noting that Lemogne used questionnaires different from MMPI. Similarly, two other studies found opposing results for repression/denial of emotions (Kune et al., 1991; Nagano et al., 2008). Kune et al. (1991) showed a positive significant association between such characteristics and incidence of CRC. More specifically, compared to healthy control subjects, cancer patients presented a higher tendency of denial and repression of negative emotions and suppression of negative emotional reactions. Indeed, Nagano et al. (2008) demonstrated no association between emotional suppression (measured with the Stress Inventory) and incidence of CRC.

Similar to Kune et al. (1991) and White et al. (2007), in a prospective study, found a weak positive association between negative affect and colorectal cancer incidence, but only after adjusting for risk factors such as dietary habits or smoking. A similar trend for anger control was present from the third year of follow-up. Regarding neuroticism, that is a trait linked to negative affect, including anger, anxiety, irritability, emotional instability (Gross, 2002) it has been measured by three studies: in the study by Kavan et al. (1995) in which MMPI-2 was used no differences were found between cancer patients and the matched healthy controls. Similarly, the study by Nakaya et al. (2003) using EPQ did not find any differences. In a later study (Nakaya et al., 2010), they confirmed that no association exists between neuroticism or any other personality characteristics (Extraversion) on incidence of CRC, using the same questionnaire in a revised version (EPI).

#### Cognitive Schemata

Cognitive schemata refer to mental structures that individuals use to organize knowledge and guide cognitive processes and behavior (Kite and Whitley, 2016). They are models built on experiences, they are stable and automatic and allow individuals to respond effortlessly to the environment and to adapt to it.

Cognitive schemata such as personality-related aspects can be represented by the factors measured by MMPI-2 (e.g., Cynicism-Normal Paranoia, Religious Fundamentalism, Dreaming, Psychotic Paranoia; Stereotypic Femininity and Masculinity) (Kavan et al., 1995). Only the Religious Fundamentalism seems to be negatively associated with CRC incidence. No association was found for Type 5 (rational anti-emotional) (Lemogne et al., 2013) and Psychoticism (Nakaya et al., 2003).

#### Relational Style

Relational style refers to the attitude people adopt when they relate to others. In particular, people naturally respond to internal and external requests. However, they may show a propensity to believe and behave with others with a self-oriented attitude or with an others-oriented attitude. Nagano et al. (2008) investigated such attitudes using the hysterical personality scale of the Stress Inventory. The hysterical personality scale is characterized by two sub-scales: egoism (a self-defensive, self-interest-oriented attitude) and object-dependence/ambivalence (oscillation between idealizing and devaluing an object or a person). The study showed a negative relationship between CRC risk and the two subscales of egoism and object-dependence/ambivalence. In this perspective, egoism and independence from others are protective factors for CRC. The importance of the focus on relationships can be seen also in Kreitler's findings (Kreitler et al., 2008): according to them, perfect duty performance, the conflict between self-effacement vs. self-assertion, and the conflict between closeness to others vs. distancing from others are the personality risk factors of colorectal cancer. Perceiving the conflict between listening and pursuing the personal needs (self-assertion) vs. the others' requests has been demonstrated as a risk factor for CRC, confirming the protective role of egoism described by Nagano et al. (2008). Extroversion (Nakaya et al., 2003), Type A (Lemogne et al., 2013), Family attachment (Kavan et al., 1995), Delinquency (Kavan et al., 1995), Social Extroversion (Kavan et al., 1995) and Inner directedness (Kavan et al., 1995) did not show significant associations with CRC incidence.

#### Body-Related Processes

Few dimensions that, in our opinion, had a lower fit with the previous categories were those related to the body as a relevant component of processes that define the way individuals feel, think and behave or interact with the self and the world. Kavan et al. (1995) used the MMPI-2 questionnaire that provides information related to the body (e.g., neurasthenic somatization or denial of somatic problems), that can be viewed as object of a possible pathology or dysfunction. No association was found between body-related factors and CRC incidence.

## Discussion and Conclusion

This systematic review showed that scant studies exist on the contribution of personality features in CRC incidence, with only eight studies reflecting the criteria of selection. Summing up the findings from the different studies, we cannot indisputably conclude that there is a data-driven role of personality in the incidence of CRC. More specifically, 4 out of the 8 analyzed studies found a positive association between the investigated personality dimensions [phobias (Kavan et al., 1995); emotional repression/denial (Kune et al., 1991); commitment to conformity to social norms (Kune et al., 1991); anger control/negative affect (White et al., 2007); conflict in self-assertion vs. self-effacement (Kreitler et al., 2008); conflict between closeness to and distance from others (Kreitler et al., 2008); aggressive hostility (Kavan et al., 1995); loss/hopelessness (Nagano et al., 2008)], perfect duty performance (Kreitler et al., 2008) and cancer incidence. Two studies found a negative association for egoism or object-dependence/ambivalence (Nagano et al., 2008) and with religious fundamentalism (Kavan et al., 1995) with CRC. The remaining 3 studies (Nakaya et al., 2003, 2010; Lemogne et al., 2013) found no association between personality dimensions and CRC incidence.

In synthesis, we can state that among personality factors the constructs of emotional regulation and relational style gathered the most part of the studies. The role of anger regulation received a consistent attention across different studies, and it might be related to CRC incidence. However, the small number of studies, the different methods of assessment and direction of the findings do not allow any clear conclusion. However, the involvement of a dysregulation of the anger control system warrant further study in the field of CRC. Anger means a cascade of events through multiple communication pathways, including autonomic and immune system, neurotransmitters, and an inflammatory cascade via the gut-brain axis potentially influencing intestinal microbiota (Huanga et al., 2021). We still do not know if long-lasting abnormal anger control may lead to gut dysbiosis-mediated inflammation which may affect colon epithelium influencing carcinogenesis (Otegbeye et al., 2021).

On the side of relational style, we note the protective role of egoism that is the only item negatively associated and it may be a topic of interest in future studies. Moreover, we underline a likely role for dimensions related to conflict aspects on the incidence of CRC. The categories of cognitive schemata and body-related processes did not reveal any relevant feature potentially related to CRC incidence. Additionally, we did not find studies outlining any significant association with categories of psychopathological meanings (e.g., neuroticism or depression).

Further studies that adopt a stronger methodology and a more homogeneous theory of personality that integrate different approaches are needed, with personality dimensions assessed over a continuum and not as simple categories (Galli et al., 2019). A topic of interest may be the study of the role of personality disorders on the CRC incidence as classified by the DSM-5 (American Psychiatric Association, 2013) or PDM (Lingiardi and McWilliams, 2017). We know that individuals with personality disorders show an increased risk of negative health outcome (Dixon-Gordon et al., 2015).

The examined studies showed lack of consistency and often contradictory findings. It was also difficult to compare results since each survey used different diagnostic tools to investigate psychological predictors. Further, studies adopted different research strategies (e.g., cohort or case-control studies, prospective or retrospective, self-assessment or clinical interviews, in-patients or subjects from national registries) and again the comparison of results was difficult. Accordingly, we need further studies adopting stronger methodology and theory of psychological functioning. Heterogeneity of results may be due to two main factors: (1) patients involved in the studies were at different stages of disease and treatment (2) different tools investigated various predictors, although similar in some cases. Difficulties of measuring personality before disease and the long time requested for such longitudinal studies could explain differences and contradictory findings. Furthermore, the study of personality should be always carried out controlling as additional covariates factors as lifestyle habits (e.g., inactivity) and nutritional factors (e.g., quantity of red/processed meat). Additionally, the inconsistency of results among studies could be explained by the absence of data on the interaction or mediation effect by other intervening variables. In this perspective, and in line with the new trends of research on big data, other psychological constructs (e.g., dispositional optimism, coping style, and self-efficacy), lifestyle habits (e.g., inactivity, smoking, nutrition), genetic and environmental factors should be investigated and analyzed together, in order to provide a more comprehensive profile of the specific cancer patient profile. The investigation of single or only a few isolated aspects that characterize the person, in his/her thinking, feeling, behavioral and relational components is one of the main shortcomings of the different studies included in the present review.

To the best of our knowledge, this is the first systematic review on the role of personality characteristics in CRC incidence, although we appreciate that our study has limitations. The definition of personality and its peculiarities is ongoing in a framework of complexity and different theoretical models, and the possibility that some studies had not been included in our review is not to be ruled out.

The prevention of CRC is a public health problem, and personalized strategies for prevention should be implemented (Oliveri et al., 2019), as modifiable risk factors are related to CRC. Furthermore, CRC incidence and mortality can be greatly reduced by screening, and again public health policies should take into account factors that may contribute to the risk of cancer, in order to tailor intervention based on specific profiles. Healthcare systems are gradually moving in the direction of personalized medicine where patients' needs, preferences and well-being are the main focus to address (Kondylakis et al., 2017; Galli and Pravettoni, 2020). The study of personality could be another key to include in the framework of any personalized approach (Gorini et al., 2015; Kondylakis et al., 2020). The development of electronic platforms that allow patients to communicate with their doctors, already allows several types of data to be gathered. This may represent an opportunity to integrate additional variables that would otherwise be difficult to collect in a single study.

## Data Availability Statement

The original contributions presented in the study are included in the article/supplementary material, further inquiries can be directed to the corresponding author/s.

## Author Contributions

The review was conceived by FG and KM. Data extraction was carried out by FG and LS with support by KM. Reporting of findings was led by FG and LS with support from SR, MGZ, KM, and GP. All authors contributed to manuscript preparation and approved it.

## Conflict of Interest

The authors declare that the research was conducted in the absence of any commercial or financial relationships that could be construed as a potential conflict of interest.

## Publisher's Note

All claims expressed in this article are solely those of the authors and do not necessarily represent those of their affiliated organizations, or those of the publisher, the editors and the reviewers. Any product that may be evaluated in this article, or claim that may be made by its manufacturer, is not guaranteed or endorsed by the publisher.
